# Treatment Decision Making at Diagnosis for Children Presenting With Advanced Cancer in Low- and Middle-Income Countries

**DOI:** 10.1200/GO-25-00320

**Published:** 2026-03-19

**Authors:** Marta Salek, Ana Caceres-Serrano, Joanne Canedo, Lucia Fuentes, Shoshana Mehler, Alaina Rule, Jamie Zeal, Carlos Rodriguez-Galindo, Dylan Graetz, Nickhill Bhakta, Erica C. Kaye, on behalf of the CATALYST Advisory Group, Justin N. Baker

**Affiliations:** ^1^ St Jude Children's Research Hospital, Memphis, TN; ^2^ Unidad Nacional de Oncologia Pediatrica, Guatemala City, Guatemala; ^3^ University of Mississippi, Oxford, MS; ^4^ University of Tennessee Health Science Center, Memphis, TN; ^5^ University of North Carolina—Chapel Hill, Chapel Hill, NC

## Abstract

**PURPOSE:**

Although most children diagnosed with cancer live in low- and middle-income countries (LMICs), research exploring decision making in these settings remains sparse. When children present with advanced cancer in LMICs, local centers may lack resources to provide treatment required to achieve cure. Existing treatment guidelines often do not account for contextual and resource variations influencing decision making. This qualitative study sought to understand physician approaches to treatment decision making for children presenting with advanced cancer at diagnosis in LMICs.

**METHODS:**

Semistructured interviews were conducted with 36 physicians caring for children with cancer across all world regions and representing diverse income levels. Interviews were conducted in English, audio-recorded, and transcribed. Inductive content analysis focused on decision-making approaches.

**RESULTS:**

Most participants were female (n = 24; 67%), older than 36 years (n = 32; 89%), and practiced at centers caring for >100 new childhood cancer cases annually (n = 26; 72%) in lower-middle–income countries (n = 20; 55%). A spectrum of cancers were reported as advanced at diagnosis, with no single diagnosis predominating. Physicians generally recommended four treatment approaches (eg, curative-intent, non–curative-intent, referral, or limited chemotherapy trial), resulting in seven outcome pathways based on whether a family accepted, challenged, or declined the proposed treatment. Four decision-making approaches (eg, physician-led, family-led, participatory, or externally influenced) informed determinations of goals of care focused on optimizing prognostic outcomes, providing individualized care, and committing to treating all children, irrespective of differences in patient/family circumstances.

**CONCLUSION:**

Physicians caring for children with cancer in LMICs navigate complex treatment decision making, considering diverse treatment paths and goals. Pragmatic, evidence-based interventions are needed to guide decision making, flexible to local constraints.

## INTRODUCTION

Treatment decision making is a core facet of high-quality pediatric cancer communication and integral to culturally adapted, patient-centered care.^1-3^ At present, most research focused on clinical decision making in pediatrics and pediatric oncology originates in high-income countries (HICs), and its application and relevance in resource-constrained or culturally diverse settings remains understudied.^4-7^ An estimated 90% of children diagnosed with cancer live in low- and middle-income countries (LMICs), yet research on decision making in these settings is sparse, with a recent review revealing only 11 studies related to decision making in pediatric cancer in LMICs.^8-10^

**CONTEXT**

**Key Objective**
This study aimed to characterize physician decision-making practices for children presenting with advanced cancer in low- and middle-income countries (LMICs), addressing a critical gap in understanding how physicians navigate treatment decisions in resource-constrained and culturally diverse settings.
**Knowledge Generated**
Physicians in 36 LMICs described diverse approaches to treatment decision making for children with advanced cancer, including both curative- and non–curative-intent therapies. They also reported varied perspectives on family involvement in decision making and treatment goals.
**Relevance**
These findings highlight the need for context-sensitive, flexible decision-making frameworks, tools, and treatment guidelines that reflect the realities of resource limitations and cultural diversity in LMICs. Applying these insights in clinical practice can help physicians tailor treatment discussions and recommendations, ultimately supporting more equitable and effective care for children presenting with advanced cancer in their settings.

Children with advanced or poor prognosis cancer at diagnosis and their families face particularly challenging circumstances around treatment decision making in LMICs as local centers often lack capacity to provide curative options or access to clinical trials.^9,11-15^ Administration of therapy misaligned with local capacity can worsen treatment-related morbidity and mortality and compromise health system efficiency and effectiveness.^11,16-18^ These challenges are further compounded by inadequate decision-making support, with existing cancer treatment guidelines anchored in data collected in HICs and focused primarily on treatment with curative intent, not accounting for varied resources and contexts.^9,17,19-30^

Further investigation of decision-making practices for children with advanced cancer in LMICs is needed to help clinicians, patients, and families make informed choices while navigating barriers to accessing first-line therapies.^7,11,12,16,31,32^ Why, when, or how physicians in LMICs decide to offer treatment with curative versus non curative intent at diagnosis and how local contextual barriers influence and complicate decision making remain poorly understood.^7,9,15^ To address this gap, this qualitative study aimed to characterize physician decision-making practices in LMICs across each of the WHO defined regions. Understanding physician decision-making approaches considerate of local contexts is an essential first step toward developing pragmatic, evidence-based resources and interventions to support decision making and improve outcomes, including survival and quality of life, for children diagnosed with advanced cancer in LMICs.

## METHODS

This study was reviewed by the Institutional Review Board at St Jude Children's Research Hospital (SJCRH) and deemed exempt in the context of deidentified data collection with physician participants. Study methods and findings are presented in accordance with the Consolidated Criteria for Reporting Qualitative Studies (COREQ) checklist.^33^

### Settings and Participants

Pediatric oncologists or pediatricians who routinely made treatment decisions for children diagnosed with cancer were eligible if they had more than 5 years of clinical experience or had completed a certified hematology/oncology fellowship, practiced in a LMIC as defined by the World Bank, and spoke English.^34^ Eligible participants were identified from the St Jude Global Alliance community, an international network of >300 medical institutions and foundations from >80 countries, dedicated to improving outcomes for children with catastrophic illness.^35^ A purposeful sample was recruited to ensure representation across diverse world regions and country income levels, with one participant invited per country. Recruitment continued until analysis of consecutive interviews yielded no new themes or insights, consistent with established standards for achieving saturation in qualitative research.^34,36,37^ Eligible participants were contacted by a member of the research team (M.S.) via e-mail, and verbal informed consent was obtained from each participant before initiating an interview.

### Study and Design

The study protocol was designed in two phases by a research team comprising experts in pediatric oncology, palliative and hospice medicine, and global health. Given the limited existing literature on decision-making approaches at diagnosis for children presenting with advanced cancer in LMICs, phase I involved the conduct of focus groups, with physicians practicing in these settings to explore current practices in up-front treatment decision making for these children. Focus group findings generated key themes to inform the development of a semistructured interview guide (Appendix Table A1) used in the second phase to investigate physician treatment decision-making approaches in greater depth.^15^ Interview prompts and probes were pilot tested with physician volunteers and iteratively revised to strengthen question prompts and probes.

Participant interviews were conducted between January 2022 and June 2023 using a secure, online videoconferencing platform that complied with data protection standards. Each interview was led by a trained expert in qualitative data collection (M.S.), lasted 30-90 minutes, and was audio-recorded. Sociodemographic data, including sex, age, country, years of clinical practice, previous training in pediatric hematology/oncology and/or palliative care, and practice setting, were collected from participants via electronic survey.

### Data Analysis

Demographic characteristics were summarized descriptively. Interview recordings were transcribed verbatim by trained medical transcriptionists and deidentified to ensure participant confidentiality. Five researchers (M.S., J.Z., A.R., J.C., S.M.) reviewed transcripts, engaged in memo writing, and conducted inductive content analysis that identified a spectrum of recurrent themes.^38^ A codebook (Appendix Table A2) was developed through iterative review of eight transcripts, and codes were applied across all transcripts by the research team (M.S., J.Z., A.R., J.C., S.M., L.F., A.C.-S.) using MAXQDA software (WERBI, Berlin, Germany). Coders met to resolve discrepancies by consensus with a third-party adjudicator (E.C.K.).

## RESULTS

Thirty-eight physicians were enrolled in the study, and 36 participants completed interviews. Demographic characteristics are summarized in Table 1. Approximately two thirds of participants were female (n = 24; 67%) and age 36-50 years (n = 22; 61%). Most practiced in a lower-middle–income country (n = 20; 55%), with participants relatively evenly distributed across WHO regions. Most physicians had more than 11 years of clinical experience (n = 28; 77%) and lacked formal pediatric hematology/oncology (n = 22; 61%) or palliative care training (n = 24; 67%). Participants worked in a variety of settings, with most employed at publicly funded hospitals (n = 16; 44%) or hospitals receiving public and private funding (n = 14; 39%). Most participants worked in settings with >100 new childhood cancer cases per year (n = 26; 72%).

**TABLE 1:** Participant Demographic Information

Characteristic	No. (%)
Age, years	
≤35	4 (11)
36-50	22 (61)
51-64	9 (25)
>65	1 (3)
Sex	
Female	24 (67)
Male	12 (33)
Country income level	
LIC	5 (14)
LMIC	20 (55)
UMIC	11 (31)
WHO region	
African	7 (19)
Eastern Mediterranean	7 (19)
European	5 (14)
Americas	7 (19)
South-East Asian	5 (14)
Western Pacific	5 (14)
Years of clinical practice since completion of medical school	
0-5	2 (6)
6-10	6 (17)
11-15	8 (22)
16-20	8 (22)
≥21	12 (33)
Completion of PHO fellowship	
Yes	30 (83)
No	6 (17)
Exposure to PHO training in a high-income country	
Yes^a^	14 (39)
No	22 (61)
Completion of training in palliative care	
Yes^b^	12 (33)
No	24 (67)
Hospital/clinic funding	
Philanthropic	4 (11)
Private	2 (6)
Public	16 (44)
Public and private	14 (39)
Yearly average of new childhood cancer cases at hospital/clinic	
≤20	1 (3)
21-49	4 (11)
50-99	5 (14)
100-299	14 (39)
≥300	12 (33)

NOTE. All participants provide cancer care for children younger than 18 years.

Abbreviations: LIC, low-income country; LMIC, lower-middle–income country; PHO, pediatric hematology-oncology; UMIC, upper-middle–income country.

^a^ For participants who had previous exposure to pediatric hematology and/or oncology training in a high-income country, two completed <1 month of training, five completed 1-6 months of training, and six completed over 7 months of training in a high-income country.

^b^ For participants who completed training in palliative care, one completed an undergraduate/medical school course, five completed a thematic or postgraduate course without official certification, and eight completed a certificate course.

### Descriptions of Advanced or Poor Prognosis Childhood Cancer

In the context of treatment decision-making approaches, participants described a spectrum of childhood cancers as advanced or poor prognosis at diagnosis. No single diagnosis was consistently described as poor prognosis by most participants. Diagnoses described by at least three participants included leukemias (AML, infant leukemia), sarcomas (Ewing, osteosarcoma, rhabdomyosarcoma), and other solid (neuroblastoma, retinoblastoma) or CNS tumors (diffuse intrinsic pontine glioma [DIPG]). Physicians' designation of advanced or poor prognosis cancers at diagnosis is presented in Table 2. In addition to naming specific diagnoses, participants also referred to categories of disease, such as advanced solid tumors or brain tumors, or nonspecific diagnoses, like metastatic sarcomas.

**TABLE 2:** Examples of Childhood Cancers at Diagnosis Considered Advanced or Associated With Poor Prognosis at Diagnosis in LMICs

Childhood Cancer	Quotation
Acute myeloid leukemia	We do not treat acute myeloid leukemia with the intention to cure it in our setting […] we make decisions honestly speaking with the family, involving them, just help them to make the decision usually to start a non-curative chemotherapy (Physician 26, LIC)
Diffuse intrinsic pontine glioma	What we offer for diffuse intrinsic pontine glioma, we give these children radiotherapy to improve quality of life and we tell the family that we give the radiation to improve the quality of life for nine months (Physician 29, LMIC)
Ewing sarcoma	We had a patient who was 14 years old with Ewing sarcoma. It took one and a half years for the child to be diagnosed in the private sector. So, it was a late referral to us, at which time he had metastasis everywhere, […] we talked to the family and we gave them an extensive explanation about the disease, what to expect and the cure rate. They accepted to be referred to our center on palliative basis (Physician 20, UMIC)
Infant leukemia	A kid with infant leukemia in our hands goes into palliative care. If we can find an opportunity outside [our home country] like going to [a medical center abroad], we seek these opportunities for these families, but that is not always possible because there is no available protocol or because the pandemic and so on (Physician 12, UMIC)
Neuroblastoma	We had a little 5-year-old boy with quite advanced neuroblastoma and he was in so much pain. The family lived very far from the hospital. We knew that we were not going to be able to cure him with the resources that we have. We sat down with the parents, and we discussed the prognosis […] We decided to give one cycle of [IV] chemotherapy […] because he looked really sick, just so we could transfer him back home and hopefully gain a little bit more time with the parents […] the patient then passed on at home, we did manage to get the patient back home alive (Physician 21, LMIC)
Osteosarcoma	I have a child with metastatic osteosarcoma with lung metastases, and treatment would include an amputation of the leg […] We know metastatic osteosarcoma carries a very poor prognosis < 20% survival rate and the family are very keen on pursuing chemotherapy but are not keen on having surgical management. If we cannot perform surgery, then we might not succeed in treating […] so our approach has moved to palliative intent (Physician 26, LMIC)
Retinoblastoma	I'm giving an example of retinoblastoma. We have bilateral retinoblastoma that is advanced stage […] we do not have access to intensive treatment to cure patients with advanced stage. If we tried to cure them with chemotherapy, we would fail, because we cannot provide optimum therapy for the high risk patient (Physician 2, LMIC)
Rhabdomyosarcoma	There will be children who will come in with stage 4 rhabdomyosarcoma. They all have effusions. They're very sick. And honestly, their general outlook is really, really poor. For those patients we will […] offer symptomatic relief, pain medication and just kind of prepare them for end of life (Physician 26, LIC)

NOTE. In addition to specific diagnoses, study participants would also refer to categories of disease, such as advanced solid tumors or brain tumors, or nonspecific diagnoses, like metastatic sarcomas. Specific diagnoses, listed in the table, appeared in a minimum of three independent participant transcripts. Additional, less commonly named specific diagnoses included atypical teratoid/rhabdoid tumor, glioblastoma, hepatoblastoma, juvenile myelomonocytic leukemia, medulloblastoma, nasopharyngeal carcinoma, non-Hodgkin lymphoma, soft tissue sarcoma, and bilateral Wilms tumor.

Abbreviations: IV, intravenous; LIC, low-income country; LMIC, lower-middle-income country; UMIC, upper-middle-income country.

### Treatment Recommendations and Outcome Pathways

**FIG 1:**
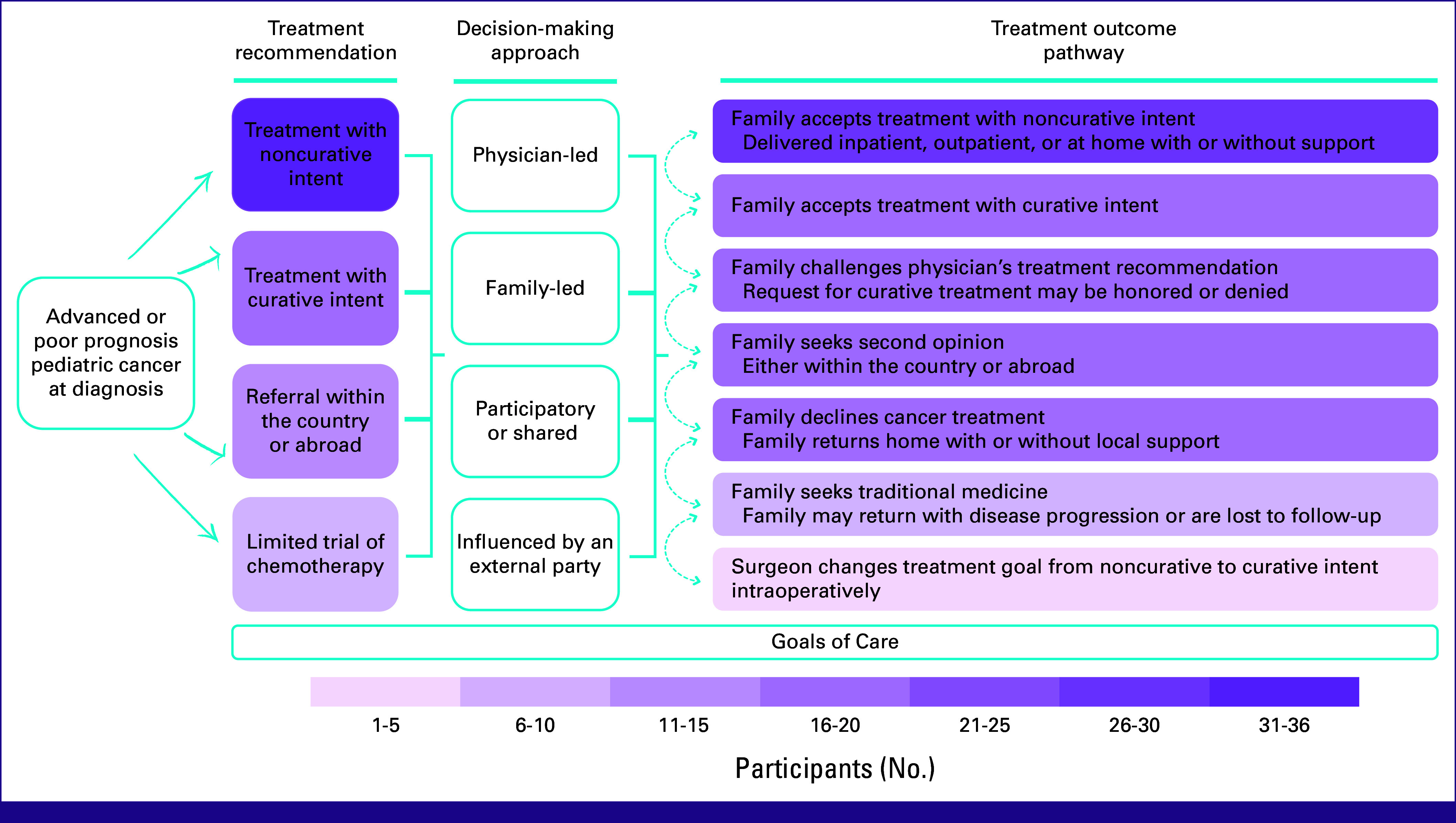
Visual summary of treatment recommendations and outcome pathways taken for children presenting with advanced or poor prognosis cancer at diagnosis. Participants described four treatment recommendations that were offered at diagnosis to children presenting with advanced cancer in their settings. This resulted in seven outcome pathways after discussion with patient's family, which did not always align with the recommendation made by the physician. Discordance between the physician's treatment recommendations and family's perspective on their child's treatment led to discussions or negotiations with their child's physician or decisions to seek care elsewhere or to decline treatment all together; this is indicated by the bidirectional arrows. In addition, one participant described changing treatment intent intraoperatively. Shading correlates with the number of participants who expressed each component of the decision-making process.

Participants described several treatment recommendations offered at diagnosis to children presenting with advanced cancer in their settings, resulting in a range of decision-making approaches, goals of care, and outcome pathways for treatment that a given patient/family might pursue which are visually presented in Figure 1. Each component is described in further detail in subsequent sections.

### Treatment Recommendations

Physicians most often recommended treatment with noncurative intent for children with advanced cancer at diagnosis, with a focus on optimizing quality of life, prolonging life, and alleviating suffering. However, physicians also described offering treatment with curative intent even in the context of very low survival odds. For example, if a child's disease had favorable clinical or diagnostic features, physicians might recommend treatment with curative intent, even if the disease burden otherwise suggested incurability. Physicians described cognitive tension between uncertainty of a child's treatment response versus known statistics associated with a given diagnosis. Some participants reported a moral and ethical obligation to treat each child with curative intent therapy regardless of diagnosis or prognosis. Others described referring patients within the country or abroad to a center with greater capacity to offer curative treatment options. Finally, some physicians described offering a limited trial of chemotherapy, at full or reduced dosing, with reassessment before transitioning to noncurative goals. Quotes illustrating these various treatment recommendations are presented in Table 3.

**TABLE 3:** Participant Quotations Related to Treatment Recommendations Offered to Children Presenting With Advanced or Poor Prognosis Cancer in LMICs

Treatment Recommendation	Quotation
Treatment with noncurative intent	I will decide case-by-case, mostly when it is advanced and incurable, and the child has comorbidities. I will decide for palliative intent [treatment] in view of the low chance of survival, and the resources that we have at hand […] mostly I [provide] palliative care with an oral metronomic regimen, but there are times where I initiate parenteral chemotherapy inpatient, for a child whom I believe may benefit for symptom relief (Physician 9, LIC)
	We consider the cancer prognosis. Even with a lot of medicine and a lot of chemotherapy, some cancers cannot be cured. We decide the tumor board meeting, [after] discussion with all consultant doctors, should we treat [the patient] with intensive chemotherapy or transfer to the palliative care unit. If the cancer prognosis and patient state is very weak, and if we have no resources [to treat the] cancer, the patient will be transferred to the palliative care unit [and receive] symptom management treatment (Physician 37, LMIC)
Treatment with curative intent	If you have a patient that you want to [advocate] that they should be treated […] even though it [would otherwise be] considered incurable, but when you look at the details, there are factors that make you [believe] the chances of survival are better, then you can make your case to the medical director […] Usually if the literature [supports your recommendation], and the multidisciplinary team agrees it is not unreasonable, they'll probably approve it (Physician 6, LMIC)
	We sometimes meet patients who are diagnosed with incurable cancer, or at least the statistics say so, and we discuss [the prognosis] with the parents. We always tell them that the survival rate is too low, less than 5%, for instance. But on the other hand, I do believe in statistics, but [children] are not just numbers. These are lives of true patients. You can never know which group the child will belong to. So, my point of view, and the point of view of my colleagues, is to start with intense treatment that is aimed to cure the patient (Physician 15, UMIC)
	We had a neuroblastoma patient that was two years old. We argued about the patient a lot because some people were saying you can't give chemotherapy because this patient has metastasis and [recommended] palliation and some were still young [and recommended] giving chemo, as it works for children under 18 months. We do not have enough research to support that it doesn't work for [children] two years old. It was emotional and we ended up [giving] chemo, curative chemo. It didn't work. It never works. The patient was better for six months but then relapsed (Physician 33, LMIC)
	We focus on the diagnosis, we focus on treating patients […] it is not an option when I received patients, that I will decide no, this [child] I will not treat and give palliative treatment. Maybe in the 80s, 90s but nowadays […] there has been an improvement in diagnosis and patients reach the hospital earlier (Physician 22, LMIC)
Limited trial of chemotherapy	Each patient and family is like a different protocol. We adapt to each family. Sometimes, we decide to go directly to metronomic therapy […] we also may discuss and decide to give two cycles at 50% [dosing] […] if the patient responds, maybe we continue. If patient doesn't respond [we pivot] to metronomic therapy (Physician 10, UMIC)
Referral within the country or abroad	Every time that we encounter a kid in that situation, we discuss the case, and sometimes we look for other opportunities outside [home country]. If that's possible we send some kids for treatment at [a medical center in a high-income country] when they have an open protocol (Physician 12, UMIC)
	For example, [for a child presenting with] acute myeloid leukemia, we ask [the family] to choose between intensive care aimed at cure or palliative care, […] intensive care aimed at cure is not provided at our hospital and patients have to travel 5 hours from our hospital to get this treatment. Most people won't travel because it's very far away (Physician 26, LIC)

Abbreviations: LIC, low-income country; LMIC, lower-middle-income country; UMIC, upper-middle-income country.

### Approaches to Decision Making

Four main approaches to decision making were described by participants, including clinician-led, family-led, participatory or shared, and decision making influenced by a party external to the physician-family dyad. Some physicians reported that they carried the burden of making treatment decisions at diagnosis on behalf of the patient and family. Physician-led decision making was described across varying clinical scenarios, for example, when only one medically reasonable option existed (eg, lack of available treatment with curative intent) or when a contextual factor, such as a physician's assessment of the family's ability to afford treatment, influenced which treatment options the physician offered to the family. Physicians also inferred that certain families preferred to have the physician guide them in decision making.

Some participants described participatory or shared approaches to decision making with the family, where the physician-family dyad would discuss the child's diagnosis and treatment options, consider the salient contextual factors affecting treatment provision, and subsequently decide together. In some contexts, families consulted with community or spiritual leaders to guide decision making. By contrast, other physicians described sharing all available treatment options, leaving treatment decision making in the hands of the family and acknowledging their inability to predict a family's wishes or available resources.

Finally, a small group of physicians described how decision making was influenced by an external group of decision-makers (eg, politicians or hospital administrators), independent of the medical team's treatment recommendations. Outcomes described by participants in these circumstances were typically poor, including risk of patient harm and moral injury to the medical team. Quotations describing various approaches to treatment recommendations are presented in Table 4.

**TABLE 4:** Participant Quotations Related to Approach to Decision Making for Children Presenting With Advanced or Poor Prognosis Cancer in LMICs

DM Approach	Quotation
Physician-led DM	Sometimes we cannot let the family decide. We have to decide ourselves. We have to be the physician [and] make a recommendation based on what you [find or see], not based on the desire of the family (Physician 10, UMIC)
	Sometimes we determine that the family [doesn't] have means or resources to even take care of themselves and cannot travel [to receive curative treatment]. So, for some [cases], in particular acute myeloid leukemia, we make the determination ahead of time [whether to share this option with the family]. Because if [you tell the family] that they could go to [the capital city to receive treatment], and they can't go, then it becomes difficult for them to stay with you [and receive treatment locally] (Physician 26, LIC)
Family-led DM	Usually, it is a discussion between the treating physician after the presentation of the case to the multi-disciplinary team, and the family, and we offer the different types of treatments and the outcomes, and then we leave the decision for the family. “Would you like to go for an aggressive treatment with little chance of success or should we start immediately palliative care?” And then at the end of the day, it's the family's decision (Physician 29, LMIC)
Participatory or shared DM	It's multifactorial. It depends on what the cancer is, how old the patient is, their functional status, the family's perspective. It's very hard to have one blanket statement for all patients with incurable cancer [… I consider] the understanding of the family, especially for younger kids more than teenagers, what the parents want, or in our culture, [the opinion of the] grandparents or the primary caregiver, what their approach is, and how aggressive they want to be. We take all of that into account before making a decision on how to move forward with treatment […] and what is in the best interest of the child (Physician 6, LMIC)
Influenced by an external party	[We had] a child who [the medical team] had all agreed we're going to treat palliatively. We discharged them with medication and they went home with a massive Wilms tumor, very aggressive. A politician brought them back to the hospital and said, “How can you neglect the child?” For them, it was neglect and then our superiors instructed us to perform surgery. We tried to push back, but they are politicians and managers of the institution. We went ahead and did the surgery. The child didn't make it through surgery because they bled out and the whole team was devastated (Physician 28, LIC)

Abbreviations: DM, decision making; LIC, low-income country; LMIC, lower-middle-income country; UMIC, upper-middle-income country.

### Goals of Care

Physicians often described the importance of optimizing prognostic outcomes for children presenting with advanced cancer. Few expressed an imperative to offer curative treatment to all children presenting with advanced disease; rather, most discussed their goal to stabilize or slow disease progression to prolong a child's life.

Diverse goals were listed under the auspices of prioritizing care of the child as an individual, with frequent references to optimizing quality of life and addressing suffering through management of pain and other cancer-related symptoms. Other common goals included maintaining a child's dignity and enabling a peaceful death. Many physicians described the importance of offering each child the best treatment possible, considering locally available resources, while minimizing treatment toxicity. At times, striving for this balance involved time-limited trials of cancer-directed treatment even when odds of cure were slim, in part to build therapeutic rapport with the family and lessen the risk of treatment abandonment or pursuit of alternative care. Physicians weighed treatment options through the lens of avoiding therapeutic cruelty (eg, not offering toxic therapies that were unlikely to achieve cure) or risking the overall health of the family (eg, anticipated financial toxicity because of staggering costs related to relocation or treatment, inability to work).

**FIG A1:**
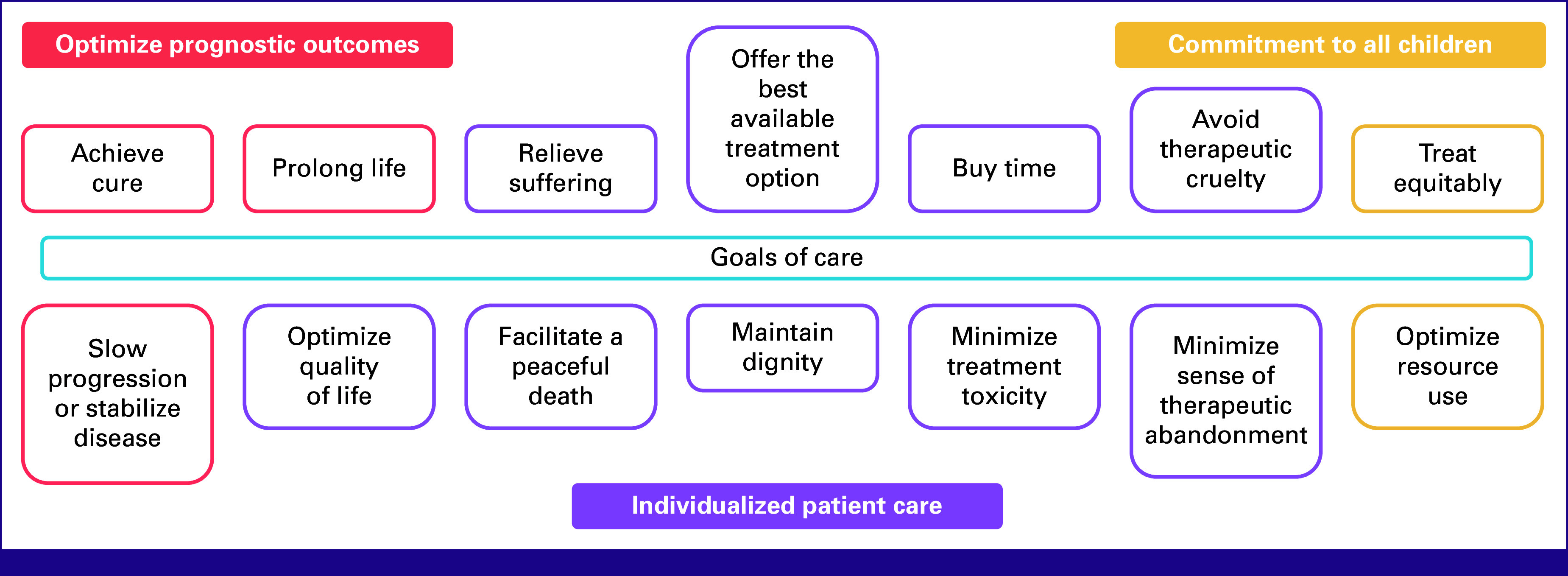
Goals of care and treatment at diagnosis for children presenting with advanced or poor prognosis cancer in low- and middle-income countries. Goals of care were grouped into three categories: optimizing prognostic outcomes, the provision of individualized patient care, and a physician's commitment to treating all children diagnosed with cancer.

Finally, participants described a profound commitment to all children diagnosed with cancer, verbalizing the importance of treating all children equally irrespective of differences in patient/family circumstances. However, participants recognized nuances within this commitment with respect to allocating limited resources to children with a higher likelihood of achieving cure. Appendix Figure A1 visually summarizes physician perspectives on goals of care, and illustrative quotes are presented in Appendix Table A3).

### Treatment Outcome Pathways

**FIG A2:**
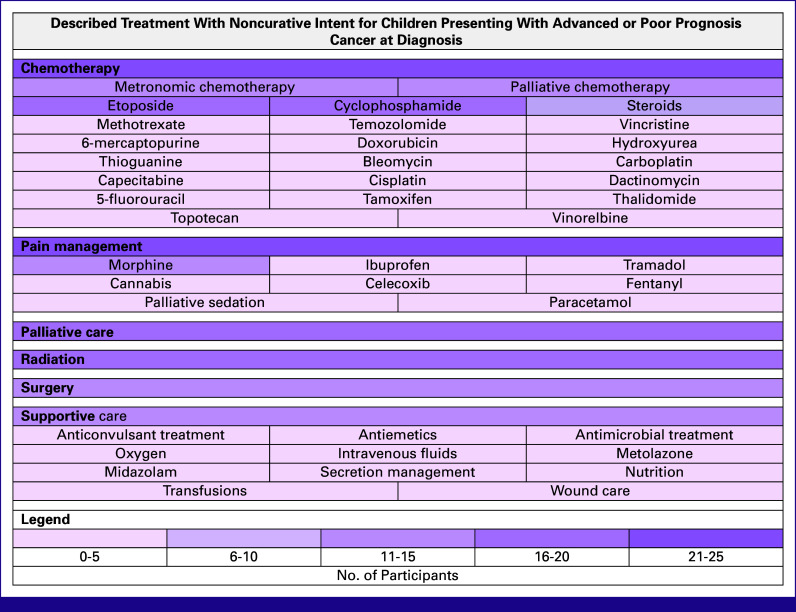
Treatments used for children diagnosed with incurable cancer at diagnosis. Participants described six groups of treatments offered up front to children diagnosed with incurable cancer. These included chemotherapy (also described as metronomic or palliative chemotherapy), pain management, palliative care, surgery, and supportive care. Specific examples described by participants (ie, etoposide) within each group rather than a general description of treatment (ie, chemotherapy) are provided. Shading correlates with legend, darker shading to indicate it was described more frequently by participants as a treatment offered.

Participants described seven potential treatment outcome pathways for children diagnosed with advanced cancer at diagnosis based on a family's response to various options (Fig 1). Quotes illustrating these treatment outcome pathways are presented in Table 5. Physicians described how families often agreed with their recommendation for noncurative treatment in inpatient, outpatient, and home settings, with specific options summarized in Appendix Figure A2. Descriptions of treatment with noncurative intent included chemotherapy (also described as metronomic or palliative chemotherapy), pain management, palliative care, radiation, surgery, and supportive care. Physicians also described families that opted for treatment with curative intent when physicians offered this option, resulting in a wide spectrum of outcomes including cure, disease control, and death from treatment toxicities.

**TABLE 5:** Participant Quotations Related to Treatment Outcome Pathways Taken by Families for Their Children, When Diagnosed With Advanced or Poor Prognosis Cancer in LMICs

Decision-Making Outcome	Quotation
Family accepts treatment with noncurative intent	There are patients that when they come [to the hospital], even the parent was discouraged. [The parent] said, “okay, they will die but do something to allow them to die comfortably (Physician 1, LMIC)
	[There are circumstances when] we have patients for whom we [gave] oral etoposide for palliative management, […] for 6 months, one year. That was 5 years ago, and now they have no disease (Physician 33, LMIC)
Family accepts treatment with curative intent	When we use intensive protocols intended for cure for these patients, we see some dying from treatment toxicity. When we see the high psychosocial and out of pocket costs and the very low probability of survival receiving the intensive protocol, we feel some discomfort, but not enough to stop discussing these options with families (Physician 8, LIC)
Family challenge recommendation for treatment with noncurative intent	If [a child presents with] neuroblastoma stage IV, advanced osteosarcoma [or] Ewing's sarcoma with metastatic features, I will definitely offer [treatment with] palliative intent because the outcome is much lower than a child with same disease in early stage […], at times families resist […], “Please do something. Take out this mass or give drugs to help them get better,” but in such scenarios we usually decide on palliative, metronomic regimens (Physician 9, LIC)
	When patients come for the first time and the disease stage is high […] most parents won't accept that the child is palliative. They want us to [try] curative [treatment…] We will try curative [treatment if] the child is in good condition to [receive] chemotherapy. If the patient is not, we tell the parents that we cannot give [curative treatment and offer] palliative therapy (Physician 1, LMIC)
	We had a 19-month-old boy with bilateral kidney cancer. The family asked for full treatment. For us, we wanted to [provide] palliative care, but the family refused […] and we started curative treatment. The child is alive now, he is three. He [received] chemotherapy, we removed the left kidney and now he is a survivor. For that reason, when a parent asks for curative treatment, even when the patient is palliative, we agree, we'll try, and we will see (Physician 1, LMIC)
Family seeks a second opinion	For rich people, they continue [seeking curative] treatment. They do not insist that we do intensive therapy, but they might not believe us. They're afraid [the cancer] could be cured in another setting. [Some may go to] a hospital in the private sector, who may tell them [the cancer] could be cured. So, they believe them […] they believe those who say it could be cured, try intensive therapy and finally the patient dies (Physician 4, LMIC)
Family declines cancer treatment (curative or non-curative intent)	We had a 12-year-old diagnosed with very late presenting lymphoma […] with deteriorating condition. After discussion with the mom, she preferred to go home because they live far and it would be logistically difficult for her to arrange the burial while the child is here [so] we prescribed [a week's supply] of meds to carry, but if that runs out, they will not have a way of getting more medication where they are going (Physician 28, LIC)
	When I explain to the family, I think that I can cure the patient, I will try to convince the family to continue with treatment. Some parents decided to withdraw. They want to take the child home (Physician 35, LMIC)
Family seeks traditional medicine	There are families that in the first meeting will say, “No, we don't want treatment, we will go to a religious [healer]. We will cure the kid” […] We talk with the family [and ask permission to] give low intensity pills or monthly chemotherapy to try to keep in touch, but the families sometimes decide to not receive anything (Physician 10, UMIC)
Physician changes the treatment goal intraoperatively	I can think of 2 or 3 patients, where the initial plan was to palliate, but then you ] change course…] it's surgical bias where you feel, “okay this tumor is too big, we're just trying to improve symptoms” […] then you end up doing a massive surgery, which you would do if the intention was curative […] it's intraoperative decision-making […] I could point [to a child] whose outcome was better than expected, but the others probably not […] the morbidity was high from surgery, they went [to the ICU]. Eventually, we lost the children, [it was not] the miraculous turnaround we expected (Physician 28, LIC)

Abbreviations: ICU, intensive care unit; LIC, low-income country; LMIC, lower-middle-income country; UMIC, upper-middle-income country.

Physicians often described tension in decision making when families challenged their recommendation for treatment with noncurative intent. Multiple scenarios were described where negotiations occurred between the family and their child's medical team. In some circumstances, physicians would explore the families' wishes but not change the offered treatment given low odds of survival. Other times, physicians changed their recommendation at the family's behest, either because the child could tolerate chemotherapy even if it was likely to be ineffective or because of potential for inaccurate prognostication.

In addition, physicians described how well-resourced families may pursue care at centers with access to more comprehensive treatment options, either guided by the physician or driven by the family independently. Participants worried about outside centers offering unrealistic chances of cure, resulting in excessive morbidity or mortality, financial exploitation, and other harms to the family. Physicians also described families that sought care from traditional healers because of cultural familiarity of offered treatments, closer proximity, or lower costs. Children were often lost to follow-up but sometimes would return in crisis with disease progression and fewer options for disease control. Some families declined treatment all together, with decisions influenced by low health literacy or socioeconomic constraints. Physicians described the logistical challenges of financing the transport of a child's body or planning a child's burial far from home, leading some families to request to return home instead of pursuing cancer-directed therapy.

Finally, one physician described a clinical scenario in which the treatment recommendation changed from noncurative to curative during the intraoperative period, because of worry that the initial prognostication was inaccurate. Retroactively, the physician reflected that the patient's odds of cure likely had not changed with treatment, and the decision to pursue a curative approach resulted in a higher risk for treatment-related morbidity and mortality.

## DISCUSSION

This qualitative study characterized treatment decision-making approaches among physicians practicing in 36 LMICs for children with advanced or poor prognosis cancer at diagnosis, revealing a diverse spectrum of treatment recommendations and pathways (Fig 1, Tables 3-5). Up-front treatment with noncurative intent was commonly endorsed although consensus was lacking on which diagnoses warranted this approach (Table 2). Physicians also had divergent perceptions of how much or often families should participate in the treatment decision-making process (Table 4). When physicians and families disagreed about treatment options, physicians reported that subsequent negotiations risked families pursuing care elsewhere or declining treatment all together (Table 5). Finally, physicians described the importance of balancing goals for up-front treatment, anchored in optimizing outcomes and offering equitable care to all children with advanced cancer (Appendix Fig A1, Appendix Table A3).

These findings suggest that approaches to up-front treatment decision making for children presenting with advanced cancer differ in LMICs compared with HICs. Of the eight cancers most often described as advanced or poor prognosis by participants, seven would be offered treatment with curative intent at diagnosis in HICs (excluding DIPG, which remains incurable globally).^39-46^ In LMICs, delays in diagnosing pediatric cancer and resource limitations often prevent centers from offering curative treatment at diagnosis to all children presenting with cancer, exacerbating outcome disparities.^11,47^ Context-sensitive approaches are needed to support decision making for children presenting with advanced cancer, empowering physicians to offer the best available treatment options—whether curative or noncurative—and identify areas for health care system strengthening. Similarly, future childhood cancer treatment guidelines should reflect local decision making realities faced in LMICs as described in this study.^9,17^ Collaborative efforts are underway to address existing guideline limitations by building on previous initiatives to develop guidelines for resource-limited settings. This includes the Adapted Resource and Implementation Application, which aims to provide pragmatic, resource-adapted guidelines tailored to local care delivery settings.^48^ Guided approaches are also needed to help support teams facing complex decision making for children with extremely low odds of cure at diagnosis, including recommendations for provision of non–curative-intent therapy or guidance on choosing between multiple reasonable treatment approaches.

Wide variations in decision-making approaches, as seen in these data, further underscore the complexity of caring for children with poor prognosis cancer in LMICs. Pediatric-focused research conducted in HICs advocates for a shared decision-making (SDM) model, an approach that is relevant when more than one medically reasonable option exists.^4,5^ In this model, clinicians and families collaborate, reviewing treatment options, preferences, and priorities. SDM was described by a subset of physicians in this study when several treatment options were felt to be reasonable at diagnosis. However, SDM is less relevant when physicians recommended only one treatment option or, conversely, perceive all options as futile. While useful in certain applications, the SDM model is not immune to bias, does not account for public health considerations (ie, resource limitations), and is anchored to Western cultural norms, suggesting the need for thoughtful consideration and exploration of how best to use this model in LMICs.^5^ A recent study exploring pediatric cancer decision making in Guatemala revealed that most families appreciated SDM while also wanting their medical team to guide decision making rather than providing multiple treatment pathways without a clear recommendation.^49^ Similar to Pakistan, families emphasized the importance of SDM and wanted to know the risks and benefits associated with treatment.^3^ Ongoing analyses are underway to better understand how contextual factors influence up-front decision making from the perspectives of children and young people presenting with advanced cancer, their families, and their multidisciplinary clinicians in LMICs. Findings from this study, along with evidence from other research on patient-centered communication, can help adapt existing decision-making frameworks developed in HICs or contribute to the creation of new models that are better suited to the unique resource and cultural contexts of LMICs.

These findings should be interpreted in the context of limitations. Data were collected from one physician per country, and study findings may not reflect the experiences of other physicians within each country. Participants were conversant in English which may exclude perspectives of non–English-speaking physicians. While not intentional, more than two thirds of participants practiced at higher-volume cancer centers; their decision-making perspectives may differ from clinicians practicing in smaller centers. As recruitment was limited to members of the St Jude Global Alliance, the participant sample may not fully represent the diversity of perspectives among physicians providing care to children diagnosed with cancer in LMICs. We sought maximum variation by including participants from diverse regions, income levels, and backgrounds. Countries were categorized by World Bank income groupings, which may obscure important differences between low-, lower-middle–, and upper-middle–income countries. In addition, 14% of participants were from low-income countries. While this reflects the global country income-level distribution according to the World Bank, it should be considered while interpreting study findings.^34^ Cultural norms and values influencing treatment decision making also vary across regions, independent of national income levels. This study was not designed to examine regional or cultural variations in-depth, representing an additional limitation. Physicians described decision-making approaches in the context of clinical vignettes rooted in their professional experiences and offered local examples of cancers considered advanced at diagnosis rather than using a standardized definition. Patient-level data were not collected to further characterize these cases. Future research should explore these distinctions as resource availability, health care infrastructure, and decision-making processes can vary substantially across LMICs. This analysis focuses on decision-making approaches; analysis of key contextual factors influencing these processes is ongoing. Finally, the perspectives of patients, caregivers, and nonphysician clinicians were not explored in this study. Future work will characterize the experiences and perspectives of these groups to complement these data and inform the design of future interventions to support clinical decision making.

In summary, physicians who care for children with advanced cancer at diagnosis in LMICs navigate complex, nuanced, context-specific treatment decision making. Although physicians aim to offer treatment with curative intent to all children presenting with cancer, current resource limitations may necessitate consideration of treatments with noncurative intent focused on relieving suffering, optimizing quality of life, and facilitating a peaceful death. Physicians face challenging decision making in their day-to-day practice as they strive to balance resource allocation with curative approaches for as many children as possible. These findings will support the development of pragmatic, evidence-based guidelines and interventions to guide treatment decision making for children presenting with advanced cancer, flexible to local constraints, and elevating the voices of key community members, including patients and families. This foundational work will also inform adaptation of existing decision-making frameworks considerate of diverse resource and cultural contexts. Ultimately, these interventions aim to improve patient outcomes and reduce suffering in synergy with efforts to optimize equitable and compassionate care for children with cancer globally.

## APPENDIX 1. CATALYST ADVISORY GROUP

Justin N. Baker, MD (Stanford University, Stanford, CA); Mae Concepcion J. Dolendo, MD, FPPS, FPSPO (Southern Philippines Medical Center, Children's Cancer Institute, Davao City, Philippines); Diego Figueredo, MD (Hospital de Clinicas, National University of Asunción, Asuncion, Paraguay); Lisa M. Force, MD, MPH (University of Washington, Seattle, WA); Paola Friedrich, MD, MPH (St Jude Children's Research Hospital, Memphis, TN); Fadhil Geriga, MBChB, MMed (Uganda Cancer Institute, Kampala, Uganda); Sanjeeva Gunasekera, MD (National Cancer Institute, Maharagama, Sri Lanka); Jean M. Hunleth, MPH, PhD (Washington University in St Louis, St Louis, MO); Roman Kizyma MD (Clinical Center of Children's Healthcare, Lviv, Ukraine); Essy Maradiege, MD (Cancer Prevention and Control Directorate - Ministry of Health, Lima, Peru); Hoa Thi Kim Nguyen, MD, PhD (Hue Central Hospital, Hue, Vietnam); Irene Nzamu, MBChB, MMed, FPHO (Kenyatta National Hospital, Nairobi, Kenya); Muhammad Rafie Raza, MD (Indus Hospital & Health Network, Karachi, Pakistan); Khilola Rustamova, MD, PhD (The Scientific and Practical Center of Pediatric Oncology, Hematology, and Immunology, Tashkent, Uzbekistan); Nur Melani Sari, MD (Dr Hasan Sadikin General Hospital/Faculty of Medicine Universitas Padjadjaran, Bandung, Indonesia); Paul H. Wise, MD, MPH (Stanford University, Stanford, CA)

**TABLE A1:** Semistructured Interview Guide

Question	Prompts
1. Think about a time when you met a new patient who was just diagnosed with advanced or incurable cancer. Can you talk me through how you decided how best to treat them? For example, did you recommend chemotherapy, radiation, surgery, other treatments, or no treatment?	a. What factors impacted your treatment recommendations? b. Can you describe who else is involved in deciding which treatment to offer? c. Is every child with the same diagnosis offered the same treatment recommendations? d. Are there guidelines, protocols, or standards of care you use to help you make decisions about which treatments to offer to children who present with advanced or incurable cancer?
2. Think about a time when you or a colleague offered treatment at the time of diagnosis that was not intended to cure the patient, rather the goal was to improve symptoms, quality of life, or to prolong life. This could include chemotherapy or radiation, or it could include simply offering medications to treat pain, nausea, or other symptoms	a. Tell me a bit about this circumstance… (without disclosing protected health information) b. What was the clinical outcome for the patient? c. At the time, did you feel comfortable (or satisfied) providing this treatment recommendation? d. What resources did you use to make this treatment recommendation (eg, guideline, colleague, IDT…)? e. Did you discuss this recommendation with colleagues before approaching the family? f. Were the patient or family part of making the decision to choose treatment that was not intended to cure? g. Did this recommendation strengthen or weaken your relationship with the patient or family? h. Does your recommendation vary if you are caring for an adolescent or young child?
3. Can you think of a time when a child presented with advanced or incurable disease and you wanted to offer treatment that focused on symptom management, quality of life, or prolonging life (eg, oral chemotherapy, or no cancer-directed treatment) but instead offered intensive treatment that is typically used when trying to cure the patient?	a. Tell me a bit about this circumstance… (without disclosing protected health information) b. What was the clinical outcome for the patient? c. What prevented you from offering treatment focused on symptoms, quality of life, or prolonging life in this situation? d. How did you feel about offering intensive treatment at the time? e. Did you discuss this recommendation with colleagues before approaching the family? f. Does this circumstance happen often? g. Were the patient or family part of making this decision? h. When you are offering chemotherapy or treatment without intention of cure what kind of treatments or medications do you offer?
4. When you do offer treatment that does not have the goal of cure, how do you describe this recommendation to patients and families?	What language or words do you use to talk about non-curative treatment options (eg, non-curative therapy, palliative therapy, supportive care, symptom management, quality of life, metronomic, treatment that does not cause added harm, etc)?
5. What resources help you make treatment decisions at diagnosis for children with advanced or incurable disease?	a. For example, guidelines, protocols, standards of care, multidisciplinary teams, tumor board, twinning programs, colleagues from other centers (national and international), other? b. Does your access to a clinical trial impact your decision making? c. What resources do not yet exist but could be helpful? d. What resources that are currently available are intended to be helpful but do not actually help in your practice?
6. Deciding on treatments for children who present with advanced or incurable disease can be difficult. How does decision making in these circumstances make you feel and/or affect you?	a. As a clinician? b. As a person? c. Does the emotional burden of decision-making impact your work? If so, how? d. What feels most challenging about deciding if or how to treat a patient diagnosed with advanced or incurable cancer?
7. Is there anything that you expected me to ask you, but I didn't ask you?	What else would you like to share about this topic?

**TABLE A2:** Codebook

Code	Definition
Physician decision making	
Approach_DM Decision-making approach of the physician	Description of the decision-making approach and recommendation of the physician, for a child presenting with advanced or incurable cancer. This reflects the decision-making process prior to discussion with the family
DxDescript_DM Descriptions of advanced or incurable cancers	Descriptions of diagnoses considered advanced or incurable that are not associated with clinical examples
NonCurativeTx_DM Examples of noncurative cancer treatment	Descriptions of management of a patient with incurable cancer, which can also be described as metronomic or palliative therapy, not associated with a clinical example, or related to supportive care
Decision making with the family	
RecMod_DMF Modification of physician's initial treatment recommendation	Modification of physician's approach or recommendation due to a factor reflective of the circumstances of the patient or family at the time of diagnosis. Distinct from routine factors physician considered when forming treatment recommendation. Also applies to choosing supportive options
SharedDM_DMF Shared decision making	Description of when the patient or family and physician contribute to the medical decision-making process to decide on treatment plan
InvolvePt_DMF Patient (child) involvement	Description of if and when the patient (child) does or does not participate in the decision-making process
Treatment decision pathway	
AcceptRec_TDO Family accepts physician's recommendation for treatment	Clinical example provided by the participant, where the family accepts the physician's recommendation for treatment
DeclineRec_TDO Family declines physician's recommendation for treatment	Clinical example provided by the participant, where the family declines the physician's recommendation for treatment and opts to return home
RequestCure_TDO Family declines physician's recommendation for noncurative treatment and requests cure	Clinical example provided by the participant, where the family declines the physician's recommendation for noncurative treatment and requests curative therapy. This also includes scenarios where the family challenges the physician's recommendation. Include clinical outcome (ie, death or cure) when coding appropriate segment
Decision-making challenges	
DM_C Challenges related to decision making	Description of the cognitive challenges experienced by the participant related to making treatment decisions for children diagnosed with advanced or incurable cancer

**TABLE A3:** Participant Quotations Related to Goals of Care and Treatment at Diagnosis for Children Presenting With Advanced or Poor Prognosis Cancer in LMICs

Treatment Goal	Quotation
Optimize prognostic outcomes	
Achieve cure	When I started my studies, multiple myeloma was an was an advanced disease [that was] not curable and the life expectancy was 12 months. When I teach my students nowadays, I tell them never to stop treatments for these patients, because we have [many] drugs and big improvements. No one should go home with palliative treatment (Physician 23, LMIC)
Slow progression or stabilize disease	[At diagnosis], we start [supportive care], but if the child doesn't die and [the clinical] condition improves, […] we can try to upgrade the level of treatment, and to give the child more chances to stay alive or lower the stage of the disease (Physician 1, LMIC)
Prolong life	We also have patients who come to us with huge tumors, a lot of metastases. We tell the parents that [the cancer] is incurable, but we will start chemo [and reassess, we do not] know for 100%. [There can be] severe worsening of the condition or the condition can improve after chemo, and we can do more to stop the disease (Physician 30, LMIC)
Individualized care of the patient and family	
Optimize quality of life	For example, we have a patient with a central nervous system tumor, and the patient is not going to cure, but we give them radiation in order to improve symptoms. We share with the family that our goal is not to cure, only to improve quality of life (Physician 3, UMIC)
Relieve suffering	When we have a patient with very advanced disease and we know that there is no possibility for them to be cured, we offer all the care that is possible to treat their symptoms. We provide oxygen. If they are in severe pain, we provide morphine […] if they are vomiting, antiemetics. If there is a case of osteosarcoma, and the child is in severe pain, there are interventions we can provide if the family agrees. For example, palliative surgery or amputation. It all depends on the illness that we are taking care of (Physician 13, LMIC)
Offer the best available treatment option	We try to do our best for every child despite the situation in which we are in, despite not having advanced machines. We cannot offer the patients the latest updates of chemotherapy, immunotherapy, or other treatments, but nevertheless, we try to give the best option which we have (Physician 5, LMIC)
Facilitate a peaceful death	I think because I learned Buddhism, the Dharma helped me a lot in terms of understanding that everyone who is born has to die. This is our final journey. If we prepare the patients to die peacefully, this is one of our goals, not only cure, to die peacefully, it is also a success (Physician 32, UMIC)
Maintain dignity	Most of the time we share the diagnosis and that there's very little chance that we are going to be able to cure this child. We are giving you these modalities of care […] the plan is that we will keep reevaluating to see how the child is doing […] I'm not sure if this will cure your child, but at least we will make sure that they have dignity as we continue (Physician 36, LMIC)
Buy time	We walk that journey with the family because [often] they have a lot of hope that when they've come to you, you're going to treat their child's disease […] but since you're trying to walk that journey, it might mean you give some treatment, to give the family time to absorb the information that there is really no chance that this child is going to survive (Physician 36, LMIC)
Minimize treatment toxicity	You have to learn to say when not to give treatment because sometimes, treatment [can lead to] more toxicity than cure or improvement […] we'd rather choose quality of life […] sometimes you're so focused on the disease that you forget that behind the disease, you are treating a child (Physician 24, LMIC)
Avoid therapeutic cruelty	Cruelty could be offering therapy when there is a low likelihood of cure [… sometimes] doctors decide to go ahead, one, three more times, without justification. Justify not in science, only for the feel that they are doing something for this patient, and don't have the capacity to stop […] The opposite situation is a modification to therapeutic effort […] to adapt the therapy with the condition of the patient. It is ethical, because it is compassionate for the patient (Physician 17, UMIC)
Minimize sense of therapeutic abandonment	[In the circumstances when] we offer palliative care, where you give one cycle, two cycles for retinoblastoma and the family sees that tumor is coming down and sharing that the treatment was just to alleviate symptoms. Then, the family doesn't feel like you've abandoned them […] because we're trying to build trust […] versus just giving paracetamol or morphine and telling them to go home […] if we've got a bit more trust, because we helped them in their time of need […] they are in a better place of acceptance (Physician 21, LMIC)
Commitment to all children	
Treat equally	Treatment for an incurable patient depends on multiple factors. If the child is dying or bedridden we give supportive care […] it also depends on the origin of the child. If the child comes from a remote place, we offer them metronomic therapy, which is easier to manage […] each patient has their story. We try to treat everyone with equality and fairness (Physician 11, LMIC)
Optimize resource use	The aim is to concentrate on children that you are likely to cure, so that those who most likely won't achieve cure do not take away the few [available] resources, to be honest. It sounds really bad, but we try to give most of the resources to children who are more likely to cure (Physician 26, LIC)

Abbreviations: LIC, low-income country; LMIC, lower-middle-income country; UMIC, upper-middle-income country.
